# Cerebrospinal fluid biomarkers provide evidence for kidney-brain axis involvement in cerebral malaria pathogenesis

**DOI:** 10.3389/fnhum.2023.1177242

**Published:** 2023-05-02

**Authors:** Andrea L. Conroy, Dibyadyuti Datta, Robert O. Opoka, Anthony Batte, Paul Bangirana, Adnan Gopinadhan, Kagan A. Mellencamp, Ayse Akcan-Arikan, Richard Idro, Chandy C. John

**Affiliations:** ^1^Ryan White Center for Pediatric Infectious Disease and Global Health, Indiana University School of Medicine, Indianapolis, IN, United States; ^2^Department of Paediatrics and Child Health, Makerere University College of Health Sciences, Kampala, Uganda; ^3^Global Health Uganda, Kampala, Uganda; ^4^Undergraduate Medical Education, The Aga Khan University, Nairobi, Kenya; ^5^Child Health and Development Centre, Makerere University College of Health Sciences, Kampala, Uganda; ^6^Department of Psychiatry, College of Health Sciences, Makerere University, Kampala, Uganda; ^7^Division of Critical Care Medicine, Department of Pediatrics, Baylor College of Medicine, Texas Children’s Hospital, Houston, TX, United States; ^8^Division of Nephrology, Department of Pediatrics, Baylor College of Medicine, Texas Children’s Hospital, Houston, TX, United States; ^9^Centre for Tropical Medicine and Global Health, University of Oxford, Oxford, United Kingdom

**Keywords:** cerebral malaria, blood-brain-barrier, cerebrospinal fluid, acute kidney injury, uremia, brain injury, pediatrics

## Abstract

**Introduction:**

Cerebral malaria is one of the most severe manifestations of malaria and is a leading cause of acquired neurodisability in African children. Recent studies suggest acute kidney injury (AKI) is a risk factor for brain injury in cerebral malaria. The present study evaluates potential mechanisms of brain injury in cerebral malaria by evaluating changes in cerebrospinal fluid measures of brain injury with respect to severe malaria complications. Specifically, we attempt to delineate mechanisms of injury focusing on blood-brain-barrier integrity and acute metabolic changes that may underlie kidney-brain crosstalk in severe malaria.

**Methods:**

We evaluated 30 cerebrospinal fluid (CSF) markers of inflammation, oxidative stress, and brain injury in 168 Ugandan children aged 18 months to 12 years hospitalized with cerebral malaria. Eligible children were infected with *Plasmodium falciparum* and had unexplained coma. Acute kidney injury (AKI) on admission was defined using the Kidney Disease: Improving Global Outcomes criteria. We further evaluated blood-brain-barrier integrity and malaria retinopathy, and electrolyte and metabolic complications in serum.

**Results:**

The mean age of children was 3.8 years (SD, 1.9) and 40.5% were female. The prevalence of AKI was 46.3% and multi-organ dysfunction was common with 76.2% of children having at least one organ system affected in addition to coma. AKI and elevated blood urea nitrogen, but not other measures of disease severity (severe coma, seizures, jaundice, acidosis), were associated with increases in CSF markers of impaired blood-brain-barrier function, neuronal injury (neuron-specific enolase, tau), excitatory neurotransmission (kynurenine), as well as altered nitric oxide bioavailability and oxidative stress (*p* < 0.05 after adjustment for multiple testing). Further evaluation of potential mechanisms suggested that AKI may mediate or be associated with CSF changes through blood-brain-barrier disruption (*p* = 0.0014), ischemic injury seen by indirect ophthalmoscopy (*p* < 0.05), altered osmolality (*p* = 0.0006) and through alterations in the amino acids transported into the brain.

**Conclusion:**

In children with cerebral malaria, there is evidence of kidney-brain injury with multiple potential pathways identified. These changes were specific to the kidney and not observed in the context of other clinical complications.

## Introduction

Severe malaria remains a leading cause of death and acquired neurologic deficits in African children (Idro et al., 2010; World Health Organization [WHO], 2021). Cerebral malaria (CM) is a form of severe malaria characterized by an infection with *Plasmodium falciparum* and coma with no other identifiable cause and requires the exclusion of meningitis, hypoglycemia and a post-ictal state as a cause for coma (World Health Organization [WHO], 2014). Despite the availability of effective antimalarial therapy to treat severe malaria (i.e., intravenous artesunate), mortality in CM remains high (Dondorp et al., 2010) and up to one-third of surviving children will experience long-term neurologic and neurocognitive consequences (Carter et al., 2005; Idro et al., 2006, 2010; John et al., 2008; Langfitt et al., 2019). The pathogenesis of cerebral malaria in children includes radiologic evidence of cerebral edema (Newton et al., 1994; Seydel et al., 2015; Sahu et al., 2021), focal blood-brain-barrier (BBB) disruption (Brown et al., 1999, 2001; Medana and Turner, 2006; Greiner et al., 2015), pronounced metabolic changes that include altered amino acid metabolism (Enwonwu et al., 1999; Lopansri et al., 2006; Gupta et al., 2017; Leopold et al., 2019; Conroy et al., 2022), acidosis (Taylor et al., 1993; Krishna et al., 1994; von Seidlein et al., 2012), and an accumulation of neuroactive metabolites (Erhardt et al., 2001; Medana et al., 2003; Holmberg et al., 2017). Pediatric CM is also characterized by a series of retinal changes that mirror findings in the brain at autopsy (Maude et al., 2009; Barrera et al., 2015), including hemorrhages, retinal whitening suggestive of tissue ischemia (Beare et al., 2009b), vessel color changes detected on indirect ophthalmoscopy, and papilledema (reviewed in Beare et al., 2004, 2009a,b; Maude et al., 2009; Barrera et al., 2015). Finally, there is emerging evidence that acute kidney injury (AKI) is an important mediator of disease severity and an independent risk factor for neurocognitive deficits and behavioral problems in survivors (Conroy et al., 2016, 2019; Namazzi et al., 2022a,b).

Acute kidney injury is defined as an abrupt loss of kidney excretory function and is diagnosed by an accumulation of nitrogenous waste products associated with reduced glomerular filtration and/or reduced urine output (Kellum et al., 2021). Two nitrogenous waste products commonly used to assess kidney function in severe malaria include creatinine, which is used to define AKI based on international consensus guidelines, and blood urea nitrogen (BUN), a less specific indicator of renal impairment (World Health Organization [WHO], 2014; Mzumara et al., 2021). AKI and BUN were independent clinical risk factors for neurologic deficits in a recent cohort of children with severe malaria (Namazzi et al., 2022a). Recent reports have also documented changes in plasma and cerebrospinal fluid (CSF) biomarkers of brain injury in the context of AKI (Datta et al., 2020, 2021). Mechanistically, studies assessing host response in AKI have highlighted endothelial and immune activation associated with AKI and mortality in children with severe malaria (Conroy et al., 2018; Ouma et al., 2020; Hawkes et al., 2022) and pronounced metabolic abnormalities in global amino acid concentrations in severe malaria-associated AKI (Conroy et al., 2022). BBB dysfunction with focal disruptions around cerebral ring hemorrhages is well described in pediatric severe malaria and correlates with retinopathy and cerebral edema (Medana and Turner, 2006; Barrera et al., 2015; Sahu et al., 2021; MacCormick et al., 2022). However, the relationship between severe malaria complications and pathways of brain injury have not been systematically evaluated.

The primary objective of this study was to evaluate the impact of AKI alongside other severe malaria complications on changes in CSF markers in children with CM. Secondary analyses explored potential mechanisms of brain injury in children with CM, focusing on loss of vascular integrity and acute electrolyte and metabolic complications.

## Materials and methods

### Study participants

Participants included children aged 18 months to 12 years enrolled at Mulago National Referral Hospital in Kampala, Uganda from 2008 to 2015 (Bangirana et al., 2014). As the primary design of the study was to evaluate cognitive outcomes, the age range of the cohort reflects the availability of validated cognitive assessment tools for the population and is inclusive of children most at risk of severe malaria. This sub-study focused on children with confirmed cerebral malaria with a Blantyre Coma Score < 3 or Glasgow Coma Score < 8 with no other identifiable cause: ruling out meningitis, a prolonged postictal state, or hypoglycemia-associated coma reversed by a glucose infusion. Exclusion criteria included prior coma, head trauma, history of hospitalization for malnutrition, cerebral palsy, or other known chronic illness (including chronic kidney disease) requiring medical care or causing developmental delay.

Children were managed according to the Uganda Clinical Guidelines at the time of the study. In the early phase of the study, intravenous quinine was the first line treatment for severe malaria given as intravenous infusion of 10 mg/kg in 5–10 ml/kg of 5% glucose over a 4-hour period, repeated every 8 h until the child could take oral medication. The parenteral phase was followed by oral quinine 10 mg/kg given three times a day to complete 7 days of treatment. Artemisinin-derivatives (artesunate or artemether) were second line therapy to be used if quinine was contraindicated or not available. In November 2012, updated Ugandan Clinical Guidelines were published recommending parenteral artesunate as the first line antimalarial for severe malaria (2.4 mg/kg for children > 20 kg or 3.0 mg/kg for children < 20 kg given at time 0, 12, and 24 h), and daily until the child could tolerate oral medication. The oral treatment was a 3-day course of artemether/lumefantrine. The change to artemisinin derivatives was implemented gradually in the health units from 2012 depending on the availability of the medicines (Conroy et al., 2021). Hypoglycemia was treated with a 1–2 ml/kg 25% dextrose bolus administered intravenously. Fluid resuscitation was managed conservatively according to local guidelines at the time of the study: a fluid bolus of 20 ml/kg of sodium chloride 0.9% intravenously over 1 h was given only for treatment of shock (systolic blood pressure <50 mmHg or absent peripheral pulse) with delayed capillary refill (> 2 s). Children without shock but with evidence of dehydration received maintenance intravenous fluids.

### Clinical definitions

To be eligible for this sub-study, all children had to have known AKI status and had a lumbar puncture with CSF stored for biomarker testing. AKI was defined using the Kidney Disease: Improving Global Outcomes (KDIGO) criteria based on a 1.5-fold increase in serum creatinine from the estimated baseline, which was defined using population-specific norms (KDIGO., 2012; Conroy et al., 2019). Elevated BUN was defined as a BUN > 20 mg/dL. Hyperlactatemia was defined as a venous lactate >5 mmol/L and severe malarial anemia was defined as a hemoglobin < 5 g/dL. Deep coma was defined as a Blantyre Coma Score (BCS) <2 or a Glasgow Coma Score (GCS) < 7. To assess BBB integrity, we calculated the CSF to plasma albumin index and categorized the presence and severity of BBB disruption using the following cut-offs: <9 (normal), 9 to < 14 (slight), 14 to < 30 (moderate), 30 or higher (severe) (Labcorp., 2021). BBB disruption was categorized as any disruption and encompassed the categories slight to severe. Plasma osmolality (posm) was calculated using the equation posm = 2(Na^+^) + (glucose/18) + BUN/2.8, where glucose and BUN are in mg/dL (Rasouli, 2016). The normal range for plasma osmolality was defined as 275 to 295 mm of solutes per one liter of plasma. Hypo-osmolality was defined as a plasma osmolality < 275 and hyper-osmolality was defined as an osmolality > 295. Multi-organ dysfunction was assessed by evaluating the number of organ systems affected based on the following: neurologic (coma), respiratory (deep breathing), hematologic (severe anemia), and hepatic (jaundice), renal (AKI).

### Study procedures

Children underwent a medical history and physical examination on enrollment. EDTA anticoagulated whole blood was collected and sent for a complete blood count to enumerate hemoglobin, platelets, and white blood cells (WBCs). This analysis was performed using Beckman Coulter ACT 5 diff hematology analyzer (Beckman Coulter Eurocenter SA, Switzerland). Malaria was assessed using standard protocols on Giemsa-stained peripheral blood smears. Biochemistries were performed on cryopreserved plasma samples for creatinine and BUN using Roche Cobas Integra 400 plus Chemistry analyzer by the Advanced Research & Diagnostic Lab at the University of Minnesota. The assay is traceable to the isotope dilution mass spectrometry (IDMS) reference standard.

**FIGURE 1:**
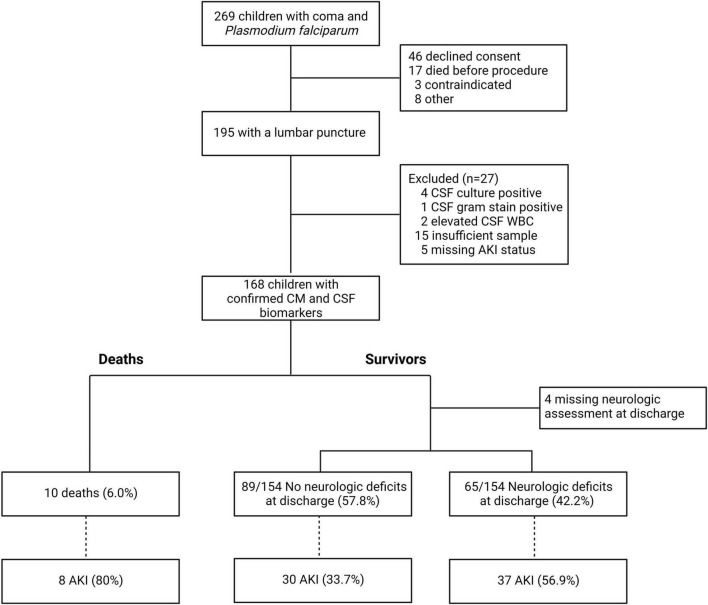
Flow diagram of study population.

Following clinical stabilization, study medical officers sought permission to do a lumbar puncture as part of routine clinical care to rule out other causes of coma. Lumbar puncture was performed unless parents or guardians did not consent to lumbar puncture (*n* = 46); the child was so ill that they died before the procedure could be done (*n* = 17); the child had contraindications to lumbar puncture, which included clinical signs of intracranial pressure, papilledema on retinal exam, or severe hemodynamic instability (*n* = 3); or for other reasons (*n* = 8, Figure 1). The procedure was conducted in a quiet, private place using aseptic technique. Samples were processed immediately with one aliquot processed for microbiology to assess cell counts, gram stain and culture, and a second aliquot stored at −80°C until testing. If immediate processing was not possible, the clinical sample was stored at room temperature for a maximum of 12 h before processing. Children were assessed for malaria retinopathy using indirect ophthalmoscopy following dilation of pupils using cyclopentolate 1% followed by tropicamide 1%. After 30 to 60 min, the retinal examination was performed by trained medical officers using a binocular indirect ophthalmoscope as previously described (Villaverde et al., 2020).

### Biomarker testing

Markers of oxidative stress included malondialdehyde (MDA) and superoxide dismutase (SOD). MDA was measured using the OxiSelect™ MDA Adduct competitive ELISA kit to detect MDA-protein adducts (Cell Biolabs Inc, San Diego, CA, USA). SOD activity and concentration were measured using a xanthine/xanthine oxidase system to generate superoxide ions (OxiSelect™ SOD activity assay, Cell Biolabs Inc, San Diego, CA, USA) and a Cu/Zn Superoxide Dismutase ELISA kit (Calbiochem, Darmstadt, Germany), respectively. Erythropoietin was measured using the Quantikine ELISA for *in vitro* diagnostic use by R&D Systems (Minneapolis, MN, USA). Cytokines, chemokines, and growth factors were assessed using the BioRad Bio-Plex magnetic bead-based multiplex assays (Hercules, CA, USA). Asymmetric dimethyl arginine (ADMA) was measured using a competitive enzyme immunoassay by Diagnostika Gmbh (Hamburg, Germany). Nitric oxide (NOx) was assessed using a fluorometric assay to measure NO2^–^ and NO3^–^ concentrations (Calbiochem, Darmstadt, Germany). Albumin was tested at the University of Minnesota Advanced Research and Diagnostic Laboratory using the Bromocresol Purple Albumin Assay (Sigma-Aldrich, St Louis, MO, USA). Neuron specific enolase (NSE) was assessed by ELISA by Alpco Diagnostics (Salem, NH, USA). Kynurenic acid (kyna) and kynurenine (kynu) were assessed using isocratic reverse-phase HPLC system, including a dual-piston, liquid delivery pump (LC 10 AD Shimadzu, Japan) and a ReproSil-Pur C18 column (4 × 100 mm, Dr. Maisch GmbH, Ammerbuch, Germany) as previously described (Holmberg et al., 2017). Tau was measured using the Luminex-based Human Tau (total) Singleplex Bead Kit (Invitrogen, Carlsbad, CA, USA) and the Human Neuroscience Buffer Reagent Kit (Invitrogen) (Datta et al., 2020).

Amino acids were measured in plasma samples by high-performance liquid chromatography using a Hitachi Amino Acid Analyzer at Fairview Lab (Minnesota, MN, USA) and quantitated relative to standards of known concentrations, as described (Conroy et al., 2022).

### Statistical analyses

Analyses were done using Stata v17.0 (StataCorp, 2021). Continuous variables were presented as mean (standard deviation) or median (interquartile range) and differences between groups were assessed using Student’s *t*-test or Wilcoxon rank sum test. Differences in proportions were compared using Pearson’s χ^2^ or Fisher’s exact test. To evaluate the relative differences in biomarker levels across clinical complications, we present data on standardized biomarkers. Biomarkers were standardized to have a mean of zero and standard deviation of one to facilitate visualization of changes within biomarkers across multiple complications and between biomarkers. A Student’s *t*-test was used to compare mean differences in standardized biomarkers across different clinical complications recognizing that multiple complications co-occur in disease. To adjust for multiple testing, Bonferroni’s correction was used adjusting for 30 biomarkers with an alpha of 0.00167. Additional linear regression models were constructed to evaluate complications independently associated with biomarker changes (Supplementary Tables 1, 2) including either AKI or BUN in the model due to collinearity. Untransformed data are presented in Supplementary Table 3. To construct forest plots, logistic regression was performed with BBB disruption, altered osmolality (hypo- or hyper-osmolality as separate categories), and retinopathy as dependent variables and clinical complications as independent variables. All regression models adjusted for age and sex. Logistic regression models evaluating the relationship between retinopathy and clinical complications also included BBB disruption and the osmolality category as covariates. Spearman correlation was used to evaluate relationships between plasma amino acid levels and CSF biomarkers adjusting for 200 comparisons with an initial alpha of *p* < 0.00025.

### Role of the funding source

The funders had no role in the study design, analysis, or decision to publish.

## Results

### Baseline characteristics and clinical complications in children with cerebral malaria

One hundred and sixty-eight children had a lumbar puncture performed with confirmed cerebral malaria and had a CSF sample stored for subsequent biomarker analysis (Figure 1). The characteristics of children with cerebral malaria who were included vs. excluded in this sub-analysis are presented in Table 1. Overall, children not included in the study (because they did not have a lumbar puncture) had similar baseline characteristics to children included other than having slightly higher weight for age z-scores (*p* = 0.014) and a lower frequency of concurrent severe malarial anemia (*p* = 0.038). Also, excluded children had a higher mortality rate (*p* < 0.001) but a lower rate of neurologic deficits (*p* = 0.006) compared to children included. This reflects the severity of illness, such that many children died before lumbar puncture could be done, and the consequent survival bias in that many of these children would likely have had neurologic deficits if they had survived.

**TABLE 1:** Children with cerebral malaria for the study.

	Included in study (*n* = 168)	Not included in the study (*n* = 101)	*P*-value
**Patient characteristics**			
Age in years	3.37 (2.56, 4.29)	3.78 (2.44, 5.24)	0.195
Sex, % female	68 (40.5)	42 (41.6)	0.858
Weight-for-age z score	−1.15 (−1.79, −0.46)	−0.78 (−1.57, −0.24)	0.021
Weight-for-height z score	−0.80 (−1.81, 0.12)	−0.55 (−1.27, 0.35)	0.072
Height-for-age z score	−1.02 (−1.98, −0.15)	−1.14 (−1.74, −0.21)	0.881
Duration of fever in days	3 (2, 4)	3 (2, 4)	0.924
HIV infection, *n* (%)	4 (2.5)	1 (1.2)	0.504
**Clinical complications**			
Coma, *n* (%)	168 (100)	101 (100)	
BCS < 2 or GCS < 7, *n* (%)	50 (29.8)	44 (44.0)	0.018
Seizures in hospital, *n* (%)	93 (55.4)	51 (50.5)	0.439
SMA, *n* (%)	43 (25.6)	15 (14.9)	0.038
Jaundice, *n* (%)	89 (53.0)	46 (45.5)	0.238
Acidosis, *n* (%)	52 (32.7)	28 (30.8)	0.752
AKI, *n* (%)	78 (46.4)	35 (38.0)	0.192
Elevated BUN, *n* (%)	66 (39.3)	29 (30.2)	0.139
**Laboratory findings**			
WBC count × 10^3^/uL	9.3 (7.2, 13.2)	9.8 (7.1, 16.2)	0.376
Hemoglobin, g/dL	6.65 (5.00, 8.65)	6.80 (5.60, 8.60)	0.411
Platelet count, × 10^3^/uL	61 (34, 101)	60 (39, 112)	0.755
Parasite density, parasites/uL	47880 (9120, 285560)	42890 (11120, 220050)	0.990
**Treatment**			
Antimalarial treatment, *n* (%)			
Quinine	116 (69.1)	70 (69.3)	0.964
Artemisinin-derivative	52 (31.0)	31 (30.7)	
Antibiotic treatment, *n* (%)	117 (69.6)	73 (72.3)	0.646
Transfusion, *n* (%)	103 (61.3)	51 (50.5)	0.083
**Outcomes**			
Mortality in-hospital, n/N (%)	10/168 (6.0)	24/101 (23.8)	<0.001
Neurologic deficit, n/N (%)	65/154 (42.2)	18/76 (23.7)	0.006

Continuous data presented as median (interquartile range) and analyzed using Wilcoxon rank sum test. Categorical data analyzed using Pearson’s Chi-square or Fisher’s exact test. BCS, Blantyre Coma Score; GCS, Glasgow Coma Score; SMA, severe malarial anemia, hemoglobin < 5 g/dL; AKI, acute kidney injury, 1.5× increase in creatinine from estimated baseline; Elevated BUN, blood urea nitrogen > 20 mg/dL; WBC, white blood cell.

The mean age of children was 3.8 years (SD, 1.9) and 40.5% were female. The mean duration of fever prior to presentation was 3.3 days (SD, 1.5). On admission, 46.4% of children (78/168) had AKI with 26.2% Stage 1 AKI, 13.1% Stage 2 AKI, and 7.1% Stage 3 AKI. The prevalence of elevated BUN was 39.3% with 73.1% (57/78) of children with AKI having elevated BUN. Other clinical complications considered included deep coma (BCS of 1 or 0, 31.8%), seizures during hospitalization (55.4%), severe malarial anemia (SMA) (25.6%), jaundice (53.0%), and hyperlactatemia (32.7%). Among the 93 children who had seizures recorded during hospitalization, 52.7% (49/93) had one seizure, 22.6% (21/93) had two, and 24.7% (23/93) had three or more seizures with a maximum of 21 recorded seizures. AKI on admission was associated with elevated BUN and SMA (*p* < 0.05) but no other complications.

### Associations of clinical complications with mortality, coma duration, and neurologic deficits

Mortality in the 168 children with CM from whom CSF was obtained was 6.0% (10/168). The presence of AKI was associated with increased odds of mortality (OR 5.03 95% CI, 1.03 to 24.44) and neurologic deficits (OR 2.60 95% CI 1.34 to 5.02) (Conroy et al., 2019). Although related to AKI, neither elevated BUN nor any of the other clinical complications (deep coma, seizures after admission, SMA, jaundice, hyperlactatemia) predicted mortality or neurologic deficits in this subset of children with CM. Complications associated with prolonged coma duration in hours included AKI (β, 95% CI: + 30.0, 19.1 to 41.6), elevated BUN (β, 95% CI: + 23.9, 12.1 to 35.7), seizures after admission (β, 95% CI: + 14.5, 3.2 to 25.9), and deep coma on admission (β, 95% CI: + 19.1, 7.2 to 31.1) (all *p* < 0.05). Elevated BUN was associated with prolonged hospitalization in days (β, 95% CI: + 1.3, 0.4 to 2.2). Among surviving children who returned for a neurologic assessment a week after hospital discharge, 42.2% had a neurologic deficit with the most common features being motor deficits (54.2%), ataxia (47.0%), abnormal reflexes (33.7%), speech deficits (32.5%), and visual deficits (19.3%).

### Blood-brain-barrier (BBB) disruption in cerebral malaria

**FIGURE 2:**
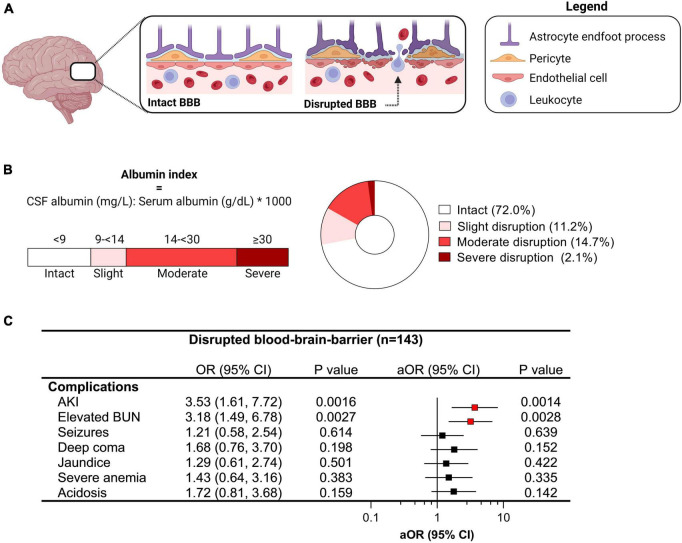
Relationship between clinical complications and blood-brain-barrier (BBB) disruption in cerebral malaria. **(A)** Diagram showing an intact and disrupted BBB characterized by increased permeability and extravasation of immune and red blood cells into the brain parenchyma due to endothelial activation. **(B)** BBB integrity was assessed using the albumin index comparing albumin levels in the cerebrospinal fluid (CSF) and plasma where the degree of BBB disruption can be quantified and categorized using established cut-offs. **(C)** Forest plot depicting the odds ratio (OR) and 95% confidence interval (CI) from logistic models were used to evaluate the relationship between clinical complications and BBB disruption (any) with adjusted OR (aOR) adjusting for age and sex. Significant relationships are depicted in red.

One of the pathways implicated in brain injury in CM is disruption of the BBB, assessed by evaluating the ratio of CSF to plasma albumin. Using established cut-offs (Labcorp., 2021), we evaluated the presence and severity of BBB disruption in children with CM with a CSF to plasma albumin ratio ≥ 9 indicative of some BBB disruption (Figure 2). Overall, 28.9% of children had evidence of BBB impairment (43/143) with 11.2% having slight disruption (16/143), 14.7% moderate disruption (21/143), and 2.1% with severe disruption (3/143) (Figure 2). Among severe malaria complications associated with impaired BBB, AKI and elevated BUN were independently associated with BBB disruption following adjustment for age and sex (Figure 2).

### Pathways of kidney-brain crosstalk in cerebral malaria

**FIGURE 3:**
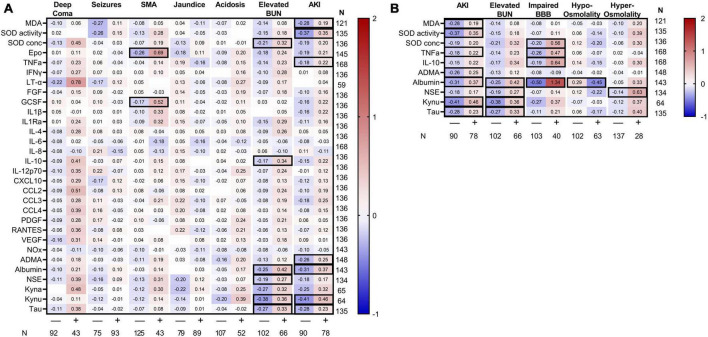
Heatmap of standardized CSF biomarkers by clinical complications. Mean standardized biomarkers are presented based on clinical complications (header on top) with the number of children with the complications within the cohort presented beneath the figure. Biomarkers are presented along the left *y*-axis with the number of samples tested presented on the right *y*-axis. **(A)** Biomarkers in clinical severe malaria complications. **(B)** Potential mechanisms of AKI-related changes in markers are depicted based on the presence of elevated BUN, impaired BBB, and changes in osmolality. Differences in standardized biomarkers were evaluated within each clinical complication using a Student’s *t*-test. Relationships significant following adjustment for multiple testing using Bonferroni correlation are outlined with a black border.

Severe malaria is characterized by multi-organ dysfunction. To delineate the contribution of severe malaria complications on changes in CSF markers, we evaluated differences in biomarkers across multiple severe malaria complications. Among all complications, AKI and elevated BUN were associated with the most pronounced differences in CSF proteins (Figure 3). Children with AKI had evidence of higher neuroinflammation (increased TNFα), neuronal injury (increased tau), excitatory neurotransmission (increased kynurenine), reduced bioavailable nitric oxide (elevated ADMA), and variable changes in markers of oxidative stress. A similar response was observed in children with elevated BUN with strong evidence of neuronal injury. Neither the depth of coma, presence of seizures, jaundice, or acidosis were associated with changes in CSF markers. However, the presence of severe anemia on admission was associated with increased erythropoietin (Epo) and granulocyte-stimulating growth factor (G-CSF) in the CSF (adjusted *p* < 0.05). Using linear regression to evaluate which complications were independently associated with CSF biomarker changes taking into consideration related complications, both SMA and jaundice were associated with increased Epo and G-CSF, while coma was associated with increased SOD concentration and LT-α in a model including AKI but not elevated BUN. The presence of AKI was associated with increased SOD activity, albumin and kynurenic acid while elevated BUN was associated with albumin, kynurenic acid, and tau (Supplementary Tables 1, 2).

### Altered plasma osmolality in cerebral malaria

**FIGURE 4:**
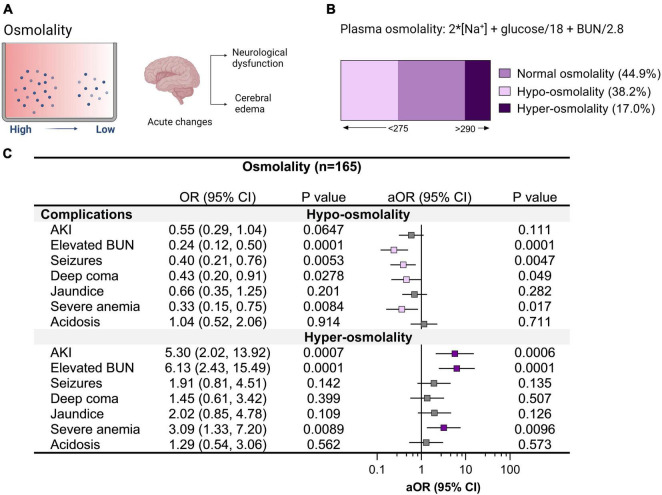
Relationship between clinical complications and altered plasma osmolality in cerebral malaria. **(A)** Plasma osmolality reflects the body’s water and electrolyte balance. The brain is sensitive to changes in plasma osmolality with acute changes in hypoosmolality linked with neurological complications and cerebral edema. **(B)** Plasma osmolality was assessed in children with cerebral malaria and categorized into hypo-osmolality (<275) and hyper-osmolality (>290) on admission to hospital using serum sodium, glucose, and blood urea nitrogen (BUN). **(C)** Forest plot showing the relationship between clinical complications and changes in osmolality using logistic regression depicting the odds ratio (OR) and adjusted OR (aOR) adjusting for child age and sex.

**FIGURE 5:**
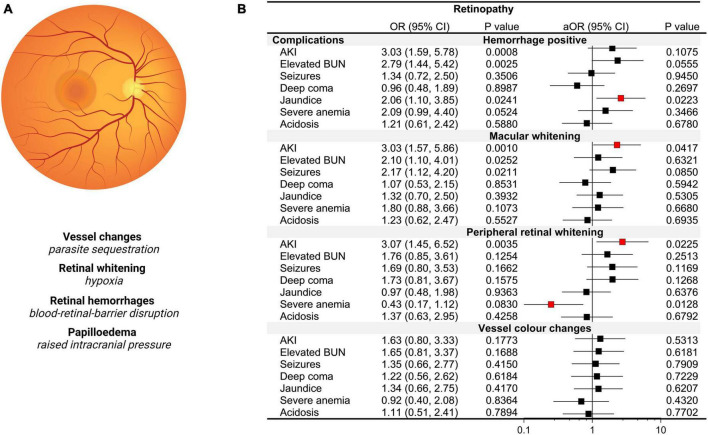
Acute kidney injury (AKI) and retinopathy based on BBB integrity and changes in plasma osmolality. **(A)** Diagram of an indirect ophthalmologic view of the retina with an accompanying description of malaria-specific retinal changes and pathological correlates. **(B)** Forest plot depicting the odds ratios (OR) and adjusted OR (aOR) from logistic regression models with the retinopathy category as the dependent variable and severe malaria complications as the independent variable. Adjusted models included age, sex, the presence of BBB disruption and osmolality category.

Cerebral edema is a common radiologic finding among children with CM (Seydel et al., 2015). Radiologic investigations into cerebral edema in pediatric CM support hypoxia-related cytotoxic edema in deep and subcortical white matter in non-fatal CM with a minority of children having vasogenic edema (Sahu et al., 2021). Over half of children had altered plasma osmolality with 38.2% hypo-osmolality and 17.0% hyper-osmolality (Figure 4). Children with hypo-osmolality at admission were less likely to have multiple seizures during hospitalization and were less likely to have retinal hemorrhages or macular whitening (Figures 4, 5). Among severe malaria complications, AKI, elevated BUN, and severe anemia were associated with hyper-osmolality (Figure 4). Although neuroimaging was not available to assess cerebral edema, malaria retinopathy was assessed (Figure 5). Among children with hyper-osmolality on admission, 11.1% (3/27) had papilledema compared to none of the children with normal or hypo-osmolality (*p* = 0.004).

### Retinopathy, the kidney, and brain injury

As the retina and brain share anatomically similar structures with the retinal vasculature considered a direct extension of cerebral vasculature, visualization of retinal changes in CM can provide insights into cerebral pathology (Maude et al., 2009). Among 164 children with retinal findings assessed, the most frequent abnormalities noted were retinal hemorrhages in 56.1% (92/164), macular whitening in 37.2% (61/164), vessel color changes in 25.0% (41/164), and peripheral whitening in 24.4% (40/164). To understand severe malaria features associated with increased risk of retinopathy, we conducted logistic regression with retinal changes as the dependent variable and ran multiple models with each severe malaria complication as a separate independent variable adjusting for age, sex, the presence of BBB disruption, and osmolality category (hypo-osmolality, normal, hyper-osmolality) (Figure 5). While AKI, elevated BUN, and jaundice were associated with retinal hemorrhages in unadjusted models, neither AKI nor elevated BUN were significant in multivariable models when adjusting for BBB disruption. AKI was a risk factor for both macular (*p* = 0.026) and peripheral whitening (*p* = 0.041) with over a threefold increase in the odds of retinal whitening (a sign of tissue ischemia) in the presence of AKI adjusting for BBB disruption, osmolality, age, and sex. Neither AKI nor any other clinical complications were associated with vessel color changes.

### Systemic metabolic complications and brain injury in cerebral malaria

Amino acid transport across the BBB is critical to support the energy needs of the brain. Recently, we reported global changes in plasma amino acid levels in CM that were associated with AKI and neurocognitive injury (Conroy et al., 2022). We hypothesized that AKI-related changes in amino acid metabolism may lead to altered levels of neuroactive metabolites. To test this hypothesis, we evaluated the relationships between plasma amino acid levels and the CSF markers altered in the context of kidney injury (Table 2). Among amino acids assessed, there were significant correlations between markers of neuronal injury (NSE, Tau) and several amino acids. In contrast, amino acids were not correlated with markers of nitric oxide bioavailability and there were weak correlations between amino acids and markers of neuroinflammation. Amino acids associated with neuronal injury included tyrosine, phenylalanine, histidine, glycine, citrulline, and asparagine. These amino acids were also associated with increased SOD concentration as an antioxidant enzyme that protects against oxidative stress but not SOD activity or MDA. Kynurenine, which is a metabolite of tryptophan metabolism, was measured in a subset of the patients (64/168) and comparison of plasma amino acids to kynurenine showed similar effect sizes to markers of neuronal injury but was not significant following adjustment for multiple testing (*p* < 0.00025).

**TABLE 2:** Spearman correlation coefficients between plasma amino acids and altered CSF markers in the context of kidney injury.

	MDA	SOD activity	SOD concentration	TNFa	IL-10	ADMA	Albumin	NSE	Kynurenine	Tau
Alanine	0.142	0.173	0.344	0.199	0.131	0.051	0.302	0.400	0.295	0.320
Arginine	0.086	0.143	0.038	0.028	−0.045	−0.018	0.142	0.120	0.082	0.091
Asparagine	−0.001	0.191	0.383	0.153	0.083	0.109	0.273	0.404	0.357	0.426
Aspartic acid	0.007	0.089	0.225	0.092	0.116	−0.022	0.125	0.104	0.184	0.225
Citrulline	0.050	0.049	0.425	0.172	0.100	0.049	0.134	0.274	0.169	0.377
Glutamine	0.172	0.250	0.323	0.084	0.125	0.088	0.312	0.332	0.190	0.439
Glycine	0.037	0.102	0.372	0.214	0.125	0.121	0.309	0.368	0.304	0.397
Histidine	0.051	0.201	0.402	0.333	0.107	0.095	0.339	0.389	0.354	0.420
Isoleucine	0.009	0.047	0.121	0.152	−0.127	0.018	0.155	0.153	−0.109	0.178
Leucine	0.006	0.240	0.163	0.182	0.048	0.042	0.321	0.292	0.255	0.317
Lysine	−0.011	0.154	0.243	0.176	−0.020	0.101	0.252	0.274	0.360	0.329
Methionine	0.045	0.105	0.345	0.197	0.086	0.001	0.273	0.337	0.328	0.401
Ornithine	0.058	0.125	0.254	0.214	−0.007	0.030	0.193	0.194	0.171	0.280
Phenylalanine	0.130	0.150	0.392	0.238	0.033	0.100	0.125	0.299	0.361	0.362
Proline	0.103	0.275	0.288	0.144	0.057	0.114	0.274	0.298	0.354	0.347
Serine	−0.010	0.143	0.173	0.046	−0.023	−0.001	0.254	0.225	0.235	0.223
Taurine	−0.036	−0.243	0.119	0.030	−0.077	−0.130	−0.061	0.103	0.000	0.167
Threonine	−0.013	0.267	0.297	0.175	0.054	0.078	0.343	0.375	0.415	0.359
Tyrosine	0.008	0.237	0.368	0.181	0.040	0.096	0.270	0.376	0.314	0.410
Valine	0.035	0.280	0.183	0.145	0.058	0.078	0.302	0.287	0.303	0.318

Spearman correlation coefficient between plasma amino acids and markers in CSF. Relationships significant after adjustment for multiple testing (200 comparisons, *p* < 0.00025) shaded. CSF, cerebrospinal fluid; MDA, malondialdehyde, SOD, superoxide dismutase, TNFα, tumor necrosis factor alpha; IL-10, interleukin 10; ADMA, asymmetric dimethyl arginine; NSE, neuron specific enolase.

## Discussion

In the present study, we showed that AKI was associated with BBB disruption, hyperosmolality, signs of retinal ischemia, and increases in markers of brain injury, and that the increases in brain injury markers correlated with global amino acid changes. These data support a growing body of evidence identifying AKI as a risk factor for neurologic deficits in severe malaria survivors and provides mechanistic insights into potential kidney-brain crosstalk pathways that may lead to brain injury in the context of AKI.

The importance of organ crosstalk is increasingly recognized as a critical determinant of both short- and long-term outcomes. The relationships between AKI and extra-renal outcomes from pediatric populations, including neurocognitive outcomes, was recently reviewed (Pande et al., 2022b). AKI is an independent risk factor for, or occurs concurrently with, neurocognitive sequelae and is evident across the spectrum of illness—from critically ill populations, to children following cardiac surgery, and community-acquired AKI (Fitzgerald et al., 2016; Myers et al., 2020; Starr et al., 2020; Pande et al., 2022a). Experimental models of AKI have demonstrated BBB disruption following short-term renal ischemia with increases in proinflammatory cytokines and chemokines in the cerebral cortex and hippocampus (central to learning and memory), measures of oxidative-stress associated cell death, astrocyte activation, and an increase in pyknotic cells (Liu et al., 2008; Chou et al., 2014). Experimental models of AKI also demonstrate functional abnormalities in the brain with accompanying motor deficits, learning problems, and memory problems (Liu et al., 2008; Firouzjaei et al., 2019). Together, preclinical findings and the growing body of clinical evidence reporting relationships between AKI and neurocognitive deficits in pediatric populations support a potential causal role for AKI in mediating brain injury.

Mechanistically, BBB disruption could permit entry of the neurotoxic metabolites into the brain resulting in neuronal injury and cell death (Lee et al., 2018). There is evidence that uremia alters sodium and calcium handling by brain cells and can impair metabolism of neurotransmitters such as acetylcholine, norepinephrine and gamma-aminobutyric acid (GABA) (Smogorzewski, 2001) which may contribute to neurological impairment in uremia. The kidney and brain are also connected via the sympathetic nervous system where activation of the reno-cerebral reflex promotes oxidative stress in the kidney and brain following ischemic injury (Cao et al., 2017). Changes in osmolality can also contribute to brain injury, and cerebral edema is well recognized in pediatric CM. In the present study, hypo-osmolality was associated with less severe neurologic changes (fewer seizures, retinal hemorrhages, and macular whitening) while hyper-osmolality was associated with the rare, but clinically important complication of papilledema. Although osmolality was driven primarily by sodium concentrations, BUN was also a contributing factor. Urea is an important osmole in both plasma and the brain that can passively diffuse across the BBB. In the present study, osmolality may reflect systemic elevations of BUN in AKI, and urea levels in the brain may mirror plasma levels. Urea transport receptor–B (UT-B) transports urea from brain cells into the CSF for excretion but is down-regulated in states of uremia (Li et al., 2012) and may exacerbate brain injury. Additional metabolomic studies are needed to evaluate uremic solutes in the plasma and CSF of children with CM.

By leveraging animal models of AKI (ischemia reperfusion injury, bilateral nephrectomy), it is possible to differentiate between AKI and uremia with or without renal ischemia on brain function (Malek, 2018). Uremia is a physiologic state characterized by the retention of a large number of compounds normally excreted by the kidney (Vanholder et al., 2003). Among known uremic solutes are BUN, ADMA, kynurenine and kynurenic acid, TNFα, IL-1β, and IL-6, all of which, in excess, can have toxic effects (Vanholder et al., 2003). In addition to being uremic toxins, kynurenic acid and kynurenine are metabolites of the tryptophan pathway and involved in excitatory neurotransmission—and an imbalance between excitatory and inhibitory neurotransmitters can impact both behavior and cognition (Stone and Darlington, 2013). Recently, we reported widespread changes in plasma amino acids in severe malaria with universal increases in plasma amino acids in AKI and evidence of plasma-related amino acid changes and long-term cognition (Conroy et al., 2022). Here we show significant correlations between several amino acids and markers of neuronal injury (NSE, Tau) and activation of an antioxidant response in the brain. The large neutral amino acid transporter (LAT1) that is highly expressed on brain endothelium preferentially transports branched amino acids (including phenylalanine, tyrosine, threonine, methionine, and histidine). LAT1 imports large and neutral amino acids in exchange for intracellular amino acids and may lead to abnormal levels of amino acid precursors for neurotransmission including tyrosine, a precursor for catecholamine and tryptophan, a precursor for serotonin (Zaragoza, 2020). The overlap in neutral amino acids with increased SOD concentration and markers of neuronal injury suggest amino acid transport may also contribute to brain injury in malaria.

Reduced nitric oxide bioavailability is well described in severe malaria (Anstey et al., 1996; Hobbs et al., 2002; Lopansri et al., 2003; Gramaglia et al., 2006; Yeo et al., 2007, 2010). Efforts to improve bioavailable nitric oxide through increased L-arginine or directly through inhaled nitric oxide have shown some benefit in improving endothelial function (Yeo et al., 2007, 2008; Serghides et al., 2011), and there is evidence from a clinical trial that inhaled nitric oxide may improve motor function following severe malaria (Bangirana et al., 2018). ADMA is a potent inhibitor of nitric oxide production that is partially cleared by the kidney. While data in the present manuscript are consistent with ADMA acting to inhibit nitric oxide synthase, we cannot exclude the possibility that it is elevated as a physiological response to inhibit local nitric oxide synthesis. Reduced nitric oxide bioavailability may be another pathway that contributes to cognitive injury in severe malaria, although to a lesser extent than other pathways (Tain and Hsu, 2017). Downregulation of urea transporter-B (UT-B) receptors which may lead to further reductions in nitric oxide production in the brain by inhibiting intracellular uptake of L-arginine and may further aggravate oxidative stress in the brain. Recently, we reported that 25% of children with AKI had a clinical indication for dialysis and there was a stepwise increase in mortality corresponding to the number of clinical indications for dialysis (Namazzi et al., 2022b). Both ADMA and SDMA are uremic toxins that are effectively cleared by dialysis (Anderstam et al., 1997). These data raise an intriguing prospect that increased access to dialysis may be beneficial in improving survival in children with severe AKI but may also help restore nitric oxide bioavailability and mitigate long-term brain injury associated with uremic toxins.

Endothelial activation is a central mediator of malaria pathogenesis and an important determinant of survival in critical illness (reviewed in Erice and Kain, 2019). In the present study, both AKI and elevated BUN were associated with significant increases of albumin in the CSF, which suggests BBB impairment. Although this study focused specifically on CSF markers, previous studies from the larger cohort have shown widespread endothelial activation associated with AKI. Specifically, increases in Angiopoietin-2, associated with increased vascular permeability and AKI, was associated with increased BBB dysfunction and worse cognitive outcomes across the age spectrum (Ouma et al., 2020, 2021). Increased BBB dysfunction associated with increased systemic endothelial activation is a likely mechanism by which uremic solutes and other host and parasite proteins cross the BBB leading to oxidative stress, inflammation, and brain injury.

While AKI was recently shown to interact with other clinical complications to increase risk of mortality in severe malaria, in the present cohort, we see that AKI and elevated BUN were associated with altered BBB function and pathways previously implicated as risk factors for long-term cognition in severe malaria survivors (Holmberg et al., 2017; Shabani et al., 2017; Datta et al., 2020). This is true even in the setting where most creatinine values were far below the WHO threshold of 3 mg/dL (World Health Organization [WHO], 2014) which may not necessarily trigger clinical concern based on clinical guidelines in malaria endemic areas. Given the susceptibility of developing brains to uremic toxins, there is a need for age-appropriate clinical cut-offs. Further studies are needed to delineate how AKI etiology, duration, severity, and recovery impact long-term neurologic recovery.

This study has several strengths including a large number of children presenting with cerebral malaria from which CSF was collected and a large number of analytes representing different pathways of potential brain injury were measured. As children often present with multiple overlapping features of severe malaria, we presented differences in CSF markers based on the presence or absence of individual severe malaria features. Alternative approaches include modeling differences based on interactions between complications but become visually more challenging to present and interpret. These data are presented in the supplement. Limitations of the study include a lack of data from children with cerebral malaria who did not have CSF obtained, either due to caregiver declining lumbar puncture or to clinical instability precluding lumbar puncture. This may affect the generalizability of the findings to all children with cerebral malaria. Radiologic investigations were not performed so we were unable to evaluate the findings with respect to cerebral edema. The integrity of the BBB and plasma osmolality were assessed on enrollment and osmolality can change rapidly in response to clinical interventions. Solutions with variable osmolality were given during the resuscitation phase (dextrose bolus, 0.9% saline) based on clinical judgment. Retinal examinations were performed following stabilization and may not reflect findings present on admission, particularly for papilledema which can be influenced by acute changes in osmolality. Despite these limitations, this is the largest cohort in which pathways of injury in CSF have been assessed, and this is the first study assess the kidney-brain axis in the context of malaria.

The present study supports a growing body of evidence for crosstalk between the kidney and brain in cerebral malaria and is consistent with results from pediatric cohorts and preclinical models identifying AKI as a key risk factor for long-term brain injury. The present study further demonstrates an association between AKI and increased oxidative stress, altered neurotransmission, neuronal injury, and reduced nitric oxide bioavailability in children with cerebral malaria. Potential mechanisms leading to CSF changes related to AKI include BBB disruption, altered osmolality, and neurotoxicity related to amino acid perturbations. Additional studies are needed to understand how the etiology and dynamics of AKI impact long-term cognitive recovery and to characterize the role of uremic toxins in cerebral malaria-related coma and long-term cognition.

## Data availability statement

The raw data supporting the conclusions of this article will be made available by the authors, without undue reservation.

## Ethics statement

Ethical approval for the study was obtained from Makerere University School of Medicine Research and Ethics Committee, the Institutional Review Board at the University of Minnesota, and the Uganda National Council for Science and Technology. Written informed consent to participate in this study was provided by the participants’ legal guardian/next of kin.

## Author contributions

CJ, RO, and RI conceptualized the study. RO, RI, PB, and CJ supervised the study conduct and were responsible for sample and data collection. AC, KM, and DD conducted the data analysis. AC wrote the first draft of the manuscript. All authors reviewed the data and substantially contributed to the analysis, interpretation of the findings, critically reviewed and edited the manuscript and approved the final version.
