# Supplementary material for: Cerebrospinal fluid biomarkers provide evidence for kidney-brain axis involvement in cerebral malaria pathogenesis
Source: Front Hum Neurosci. 2023 May 2;17:1177242. doi: 10.3389/fnhum.2023.1177242 (PMC10185839; doi:10.3389/fnhum.2023.1177242)
Supplement: Supplementary file 1 [file Table_1.docx]

**Supplemental Table 1. Adjusted β coefficients from multiple linear regressions predicting standardized biomarker levels**

|  | **Complications** | | | | | |  |
| --- | --- | --- | --- | --- | --- | --- | --- |
| **Biomarker** | **Deep Coma** | **Seizures** | **SMA** | **Jaundice** | **Acidosis** | **AKI** | **N** |
| MDA | 0.11 | 0.35 | 0.08 | -0.29 | 0.07 | 0.40 | 119 |
| SOD activity | -0.23 | 0.40 | 0.26 | -0.25 | -0.06 | 0.64 | 132 |
| SOD conc | 0.62 | -0.06 | 0.11 | 0.19 | -0.06 | 0.30 | 133 |
| Epo | 0.23 | -0.18 | 0.95 | 0.01 | 0.22 | 0.16 | 139 |
| TNFa | 0.22 | -0.14 | 0.28 | -0.50 | 0.18 | 0.37 | 159 |
| IFNγ | 0.34 | -0.10 | 0.15 | -0.02 | 0.20 | 0.02 | 133 |
| LT-α | 0.94 | 0.24 | -0.07 | -0.31 | 0.34 | 0.07 | 58 |
| FGF | 0.09 | -0.16 | 0.01 | -0.14 | 0.25 | 0.33 | 133 |
| GCSF | -0.13 | -0.10 | 0.67 | -0.46 | 0.03 | 0.24 | 133 |
| IL1β | -0.18 | -0.01 | 0.43 | -0.31 | -0.02 | 0.34 | 133 |
| IL1Ra | 0.21 | -0.05 | 0.33 | -0.28 | 0.12 | 0.09 | 133 |
| IL-4 | 0.42 | -0.16 | -0.01 | 0.06 | 0.11 | -0.02 | 133 |
| IL-6 | 0.02 | -0.02 | -0.13 | -0.19 | -0.08 | 0.10 | 159 |
| IL-8 | 0.42 | -0.42 | -0.00 | -0.25 | 0.01 | -0.23 | 133 |
| IL-10 | 0.35 | 0.04 | 0.13 | -0.09 | -0.04 | 0.30 | 133 |
| IL-12p70 | 0.48 | -0.38 | 0.24 | -0.23 | 0.33 | 0.05 | 133 |
| CXCL10 | 0.17 | 0.29 | 0.12 | -0.33 | 0.06 | 0.15 | 133 |
| CCL2 | 0.45 | 0.10 | -0.14 | -0.07 | 0.05 | 0.16 | 133 |
| CCL3 | 0.23 | -0.19 | 0.32 | -0.43 | -0.15 | 0.36 | 133 |
| CCL4 | 0.41 | -0.26 | 0.03 | -0.33 | 0.04 | 0.15 | 133 |
| PDGF | 0.42 | -0.27 | -0.04 | 0.02 | 0.33 | -0.02 | 133 |
| RANTES | 0.32 | 0.13 | 0.07 | -0.36 | 0.28 | 0.08 | 133 |
| VEGF | 0.52 | -0.24 | 0.10 | 0.14 | 0.16 | -0.13 | 133 |
| NOx | -0.05 | 0.04 | 0.06 | -0.09 | -0.01 | 0.01 | 139 |
| ADMA | 0.45 | -0.08 | 0.24 | -0.19 | 0.34 | 0.43 | 142 |
| Albumin | 0.21 | 0.13 | 0.04 | -0.01 | 0.16 | 0.66 | 140 |
| NSE | 0.39 | 0.18 | 0.33 | 0.17 | 0.05 | 0.22 | 131 |
| Kyna | 0.60 | -0.13 | 0.01 | 0.20 | 0.21 | 0.62 | 61 |
| Kynu | 0.20 | 0.03 | -0.04 | -0.01 | 0.49 | 0.89 | 60 |
| Tau | 0.40 | -0.08 | -0.07 | 0.07 | -0.05 | 0.43 | 132 |
| Each row represents a multiple linear regression. Biomarkers in each row were regressed on all complications plus age and sex. Significant coefficients after adjustment for multiple comparisons (8 predictors, p<0.00625) shaded. | | | | | | | |
