# Supplementary material for: Cerebrospinal fluid biomarkers provide evidence for kidney-brain axis involvement in cerebral malaria pathogenesis
Source: Front Hum Neurosci. 2023 May 2;17:1177242. doi: 10.3389/fnhum.2023.1177242 (PMC10185839; doi:10.3389/fnhum.2023.1177242)
Supplement: Supplementary file 3 [file Table_3.docx]

**Supplemental Table 3. Untransformed biomarker levels according to the presence of clinical complications**

|  |  | **Complications** | | | | | | | | | | | | | |  |
| --- | --- | --- | --- | --- | --- | --- | --- | --- | --- | --- | --- | --- | --- | --- | --- | --- |
|  | **Deep Coma** | | | **Seizures** | | **SMA** | | **Jaundice** | | **Acidosis** | | **Elevated BUN** | | **AKI** | | |
| **Biomarker** | **Absent** | | **Present** | **Absent** | **Present** | **Absent** | **Present** | **Absent** | **Present** | **Absent** | **Present** | **Absent** | **Present** | **Absent** | **Present** | |
| MDA (umol/l) | 0.34 (0.27, 0.42) | | 0.37 (0.27, 0.49) | 0.35 (0.26, 0.41) | 0.34 (0.28, 0.49) | 0.34 (0.26, 0.45) | 0.37 (0.31, 0.45) | 0.36 (0.29, 0.48) | 0.34 (0.27, 0.43) | 0.33 (0.27, 0.42) | 0.38 (0.29, 0.47) | 0.34 (0.27, 0.44) | 0.35 (0.27, 0.48) | 0.31 (0.26, 0.39) | 0.39 (0.31, 0.50) | |
| SOD activity (mU/ul) | 102.62 (54.55, 162.38) | | 113.73 (54.72, 164.39) | 71.72 (49.27, 146.08) | 132.69 (62.18, 166.51) | 96.95 (53.32, 159.13) | 137.94 (70.12, 178.46) | 124.12 (53.39, 160.84) | 94.27 (55.42, 162.75) | 101.29 (52.66, 162.38) | 108.39 (67.53, 163.11) | 98.20 (52.44, 151.31) | 135.31 (59.61, 178.55) | 62.36 (46.07, 124.12) | 146.08 (81.66, 178.55) | |
| SOD conc (ng/mL) | 76.50 (51.80, 108.20) | | 123.40 (59.80, 173.60) | 92.40 (67.00, 121.80) | 75.70 (48.20, 125.10) | 73.00 (50.00, 114.20) | 92.40 (75.20, 158.00) | 66.00 (48.80, 124.80) | 90.80 (68.00, 122.00) | 76.20 (51.80, 116.60) | 96.90 (61.20, 152.00) | 69.60 (49.80, 97.60) | 109.70 (71.40, 155.60) | 72.10 (50.00, 106.60) | 92.30 (59.80, 154.80) | |
| Epo  (mU/ul) | 8.65 (4.20, 18.83) | | 7.10 (3.31, 16.36) | 9.01 (3.78, 17.43) | 7.38 (4.14, 17.89) | 6.42 (3.35, 13.19) | 19.13 (7.35, 44.56) | 6.39 (3.31, 13.42) | 10.31 (5.27, 20.12) | 8.21 (3.90, 14.88) | 9.70 (4.14, 20.22) | 6.66 (3.50, 13.19) | 12.25 (6.21, 22.19) | 6.80 (3.50, 11.84) | 12.98 (5.22, 24.50) | |
| TNFa | 1.29 (0.44, 2.73) | | 2.08 (0.96, 4.83) | 1.61 (0.59, 3.25) | 1.20 (0.55, 3.16) | 1.46 (0.76, 2.87) | 1.12 (0.06, 3.96) | 1.49 (0.76, 4.83) | 1.41 (0.38, 2.87) | 1.20 (0.55, 2.73) | 1.85 (0.62, 4.63) | 1.29 (0.44, 2.53) | 2.03 (0.76, 4.93) | 1.35 (0.65, 2.58) | 1.79 (0.39, 4.93) | |
| IFNγ | 22.01 (11.57, 34.29) | | 24.16 (19.07, 38.23) | 23.37 (11.57, 36.26) | 22.07 (14.63, 36.67) | 22.07 (14.63, 37.07) | 23.37 (14.63, 34.29) | 22.07 (14.63, 31.69) | 23.37 (11.57, 38.23) | 22.07 (14.63, 33.05) | 23.37 (14.63, 38.23) | 21.71 (14.63, 30.29) | 24.36 (14.63, 38.23) | 22.07 (14.63, 38.23) | 23.37 (14.63, 31.69) | |
| LT-α | 0.62 (0.62, 0.62) | | 0.62 (0.62, 1.45) | 0.62 (0.62, 0.62) | 0.62 (0.62, 0.62) | 0.62 (0.62, 0.62) | 0.62 (0.62, 0.62) | 0.62 (0.62, 0.62) | 0.62 (0.62, 0.62) | 0.62 (0.62, 0.62) | 0.62 (0.62, 0.62) | 0.62 (0.62, 0.62) | 0.62 (0.62, 0.62) | 0.62 (0.62, 0.62) | 0.62 (0.62, 0.62) | |
| FGF | 4.55 (2.78, 11.66) | | 4.55 (4.55, 11.66) | 4.55 (4.55, 12.19) | 4.55 (2.78, 11.34) | 4.55 (4.55, 11.66) | 4.55 (2.78, 11.03) | 4.55 (4.55, 15.75) | 4.55 (4.55, 4.55) | 4.55 (4.55, 11.12) | 4.55 (2.78, 17.66) | 4.55 (4.55, 11.66) | 4.55 (2.78, 13.75) | 4.55 (4.55, 4.55) | 4.55 (2.78, 13.24) | |
| GCSF | 39.34 (22.18, 115.78) | | 68.56 (32.95, 142.29) | 44.47 (25.36, 125.84) | 53.68 (22.62, 124.59) | 37.84 (22.18, 90.21) | 94.40 (31.50, 273.38) | 49.74 (22.18, 175.95) | 49.56 (24.52, 95.51) | 39.15 (21.39, 88.22) | 91.01 (29.95, 175.95) | 36.97 (22.18, 104.17) | 73.42 (30.16, 135.15) | 32.23 (21.39, 86.76) | 74.89 (30.16, 142.29) | |
| IL1β | 0.48 (0.48, 0.48) | | 0.48 (0.48, 0.48) | 0.48 (0.48, 0.48) | 0.48 (0.48, 0.48) | 0.48 (0.48, 0.48) | 0.48 (0.48, 1.91) | 0.48 (0.48, 0.48) | 0.48 (0.48, 0.48) | 0.48 (0.48, 0.48) | 0.48 (0.48, 0.48) | 0.48 (0.48, 0.48) | 0.48 (0.48, 2.00) | 0.48 (0.48, 0.48) | 0.48 (0.48, 2.00) | |
| IL1Ra | 49.69 (33.61, 79.64) | | 54.35 (28.13, 109.54) | 48.80 (31.73, 71.34) | 55.30 (28.99, 100.13) | 48.80 (29.85, 80.90) | 60.02 (29.85, 117.20) | 59.10 (37.37, 105.71) | 48.80 (29.85, 79.00) | 48.80 (28.13, 79.64) | 69.13 (33.61, 100.13) | 41.13 (29.85, 69.37) | 68.48 (39.25, 110.35) | 40.19 (29.85, 69.37) | 68.24 (38.48, 100.13) | |
| IL-4 | 0.41 (0.27, 0.78) | | 0.62 (0.35, 0.84) | 0.49 (0.29, 0.84) | 0.44 (0.28, 0.81) | 0.52 (0.29, 0.84) | 0.35 (0.26, 0.84) | 0.44 (0.29, 0.84) | 0.52 (0.26, 0.84) | 0.44 (0.29, 0.84) | 0.57 (0.26, 0.84) | 0.44 (0.29, 0.62) | 0.57 (0.26, 0.99) | 0.57 (0.35, 0.84) | 0.35 (0.21, 0.84) | |
| IL-6 | 9.96 (3.84, 20.10) | | 20.82 (6.35, 35.46) | 12.06 (3.87, 29.88) | 11.43 (4.01, 24.02) | 13.13 (3.93, 34.03) | 9.75 (3.78, 15.99) | 19.02 (4.53, 39.06) | 9.75 (3.57, 15.99) | 10.68 (3.84, 22.69) | 13.79 (4.63, 29.70) | 11.36 (3.87, 25.90) | 12.29 (5.13, 34.03) | 10.54 (3.84, 26.39) | 12.75 (5.13, 30.34) | |
| IL-8 | 584.77 (312.10, 1275.87) | | 654.39 (458.75, 1368.74) | 866.38 (316.36, 1942.77) | 560.70 (329.62, 1004.91) | 693.36 (325.07, 1287.62) | 463.84 (315.00, 1489.16) | 748.75 (458.75, 1470.92) | 463.84 (261.84, 1185.67) | 571.41 (279.56, 1185.67) | 704.40 (347.13, 1512.62) | 627.78 (326.72, 1275.87) | 615.99 (223.68, 1312.88) | 553.80 (317.71, 1512.62) | 655.28 (315.00, 1145.90) | |
| IL-10 | 5.02 (3.53, 8.33) | | 5.25 (3.56, 15.33) | 4.90 (3.45, 7.89) | 5.25 (3.54, 12.20) | 5.06 (3.45, 8.33) | 6.04 (3.56, 17.63) | 5.13 (3.56, 13.03) | 5.02 (3.46, 8.10) | 4.74 (3.23, 8.46) | 6.15 (4.01, 11.89) | 4.74 (3.53, 7.98) | 6.15 (3.56, 15.33) | 5.02 (3.53, 7.87) | 5.50 (3.53, 13.03) | |
| IL-12p70 | 3.91 (1.85, 5.83) | | 4.58 (3.35, 7.50) | 3.91 (2.51, 8.34) | 4.32 (2.29, 5.36) | 3.94 (2.41, 6.29) | 4.28 (2.17, 6.76) | 4.75 (2.91, 6.29) | 3.91 (1.85, 6.29) | 3.91 (1.85, 5.83) | 4.47 (2.65, 8.57) | 3.82 (2.38, 5.21) | 4.75 (2.41, 7.67) | 3.91 (2.38, 6.29) | 4.32 (2.41, 5.83) | |
| CXCL10 | 2694.97 (1626.14, 6504.61) | | 5018.70 (2036.64, 14062.59) | 2471.30 (1494.57, 5583.52) | 3279.11 (1795.46, 9155.47) | 2568.14 (1626.14, 7501.41) | 4446.72 (2118.52, 6569.76) | 2816.57 (1860.40, 9005.00) | 2742.11 (1442.97, 6957.60) | 2475.40 (1550.81, 6822.59) | 4092.76 (2280.42, 9005.00) | 2609.39 (1442.97, 6234.87) | 3724.11 (1831.47, 10184.31) | 2381.27 (1364.74, 6822.59) | 3536.75 (1859.58, 8696.61) | |
| CCL2 | 572.61 (325.11, 1031.41) | | 980.67 (486.34, 2042.75) | 592.70 (358.18, 1007.79) | 721.82 (333.26, 1251.65) | 661.56 (325.11, 1273.32) | 562.81 (405.08, 941.12) | 678.87 (369.96, 1303.61) | 616.70 (286.30, 1099.47) | 675.98 (286.30, 1094.26) | 614.48 (443.74, 1229.98) | 588.96 (355.03, 1008.86) | 889.16 (337.67, 1273.32) | 606.36 (325.11, 1031.41) | 761.90 (355.03, 1292.65) | |
| CCL3 | 2.21 (1.23, 4.06) | | 2.70 (1.85, 5.30) | 2.39 (1.31, 4.13) | 2.39 (1.45, 4.52) | 2.39 (0.35, 4.21) | 2.32 (1.68, 4.63) | 2.46 (1.63, 4.63) | 2.08 (1.40, 4.02) | 2.25 (1.23, 4.49) | 2.56 (1.50, 4.21) | 2.21 (1.40, 3.86) | 2.53 (1.23, 4.63) | 2.13 (1.40, 3.18) | 2.65 (1.23, 4.75) | |
| CCL4 | 49.43 (33.04, 64.76) | | 52.86 (40.82, 96.38) | 48.64 (33.84, 68.96) | 52.87 (38.24, 67.22) | 50.56 (34.64, 68.00) | 49.57 (37.15, 63.05) | 47.13 (34.96, 69.04) | 52.38 (34.76, 66.96) | 49.29 (34.64, 67.09) | 50.98 (34.92, 69.04) | 50.06 (34.96, 63.05) | 50.41 (34.76, 73.30) | 49.43 (37.15, 63.05) | 52.80 (32.17, 75.73) | |
| PDGF | 7.71 (0.15, 12.91) | | 9.36 (4.12, 14.70) | 8.92 (0.15, 14.03) | 7.93 (0.15, 12.91) | 8.48 (0.15, 13.80) | 7.19 (0.15, 12.91) | 7.19 (0.15, 14.03) | 8.48 (2.14, 13.36) | 7.60 (0.15, 12.02) | 9.14 (0.15, 14.70) | 7.71 (0.15, 12.91) | 8.46 (0.15, 14.03) | 8.48 (5.85, 13.36) | 7.24 (0.15, 13.80) | |
| RANTES | 9.38 (6.54, 14.34) | | 9.14 (7.90, 16.50) | 9.16 (6.54, 13.65) | 9.36 (7.86, 15.83) | 9.14 (6.85, 14.34) | 10.77 (6.54, 14.56) | 9.85 (7.90, 15.83) | 8.95 (6.54, 12.93) | 8.95 (6.54, 13.12) | 10.65 (7.90, 15.83) | 9.04 (6.58, 13.34) | 9.85 (6.85, 16.68) | 8.95 (6.54, 13.14) | 10.54 (7.81, 16.50) | |
| VEGF | 16.63 (9.14, 23.25) | | 21.09 (10.75, 31.52) | 16.66 (10.14, 28.48) | 16.93 (9.14, 24.82) | 16.69 (9.14, 26.27) | 15.69 (10.07, 26.41) | 15.69 (9.14, 26.00) | 19.42 (9.68, 26.55) | 16.63 (9.83, 26.00) | 17.40 (9.59, 31.52) | 15.34 (9.60, 23.25) | 19.77 (10.07, 29.86) | 17.64 (9.83, 30.41) | 15.38 (9.14, 26.00) | |
| NOx (umol/l) | 0.73 (0.11, 2.49) | | 0.49 (0.02, 1.95) | 0.51 (0.02, 1.90) | 0.86 (0.16, 2.95) | 0.50 (0.02, 1.68) | 1.25 (0.39, 4.57) | 0.73 (0.02, 2.49) | 0.71 (0.11, 2.23) | 0.71 (0.13, 2.32) | 0.78 (0.06, 3.34) | 0.49 (0.02, 1.90) | 1.08 (0.22, 3.66) | 0.40 (0.02, 1.36) | 1.24 (0.30, 3.40) | |
| ADMA (umol/l) | 0.11 (0.08, 0.14) | | 0.14 (0.11, 0.17) | 0.12 (0.09, 0.15) | 0.12 (0.09, 0.15) | 0.12 (0.09, 0.15) | 0.13 (0.09, 0.18) | 0.13 (0.09, 0.15) | 0.12 (0.09, 0.15) | 0.12 (0.09, 0.14) | 0.13 (0.10, 0.17) | 0.12 (0.09, 0.15) | 0.13 (0.10, 0.17) | 0.12 (0.08, 0.14) | 0.13 (0.10, 0.17) | |
| Albumin  (mg/L) | 137.50 (84.50, 243.00) | | 155.00 (95.00, 337.00) | 118.50 (86.00, 256.00) | 162.00 (95.00, 302.00) | 140.00 (85.00, 250.00) | 169.00 (106.00, 337.00) | 141.50 (94.00, 212.00) | 150.00 (83.00, 285.00) | 144.00 (86.00, 244.00) | 150.00 (84.00, 329.00) | 112.50 (79.00, 183.00) | 204.00 (119.00, 368.00) | 114.50 (79.00, 179.00) | 201.00 (100.00, 367.00) | |
| NSE (ng/mL) | 2.29 (1.52, 4.40) | | 3.44 (1.66, 7.58) | 2.15 (1.43, 4.50) | 2.66 (1.65, 5.04) | 2.59 (1.57, 4.44) | 2.35 (1.54, 4.84) | 2.33 (1.29, 4.40) | 2.75 (1.63, 4.99) | 2.27 (1.43, 4.40) | 3.24 (2.04, 5.14) | 2.01 (1.43, 3.67) | 3.15 (2.15, 5.46) | 1.93 (1.43, 3.67) | 3.12 (2.02, 5.18) | |
| Kyna  (mM) | 221.94 (137.48, 407.89) | | 331.75 (106.78, 451.21) | 239.99 (136.83, 524.58) | 270.55 (126.44, 419.44) | 238.20 (109.44, 407.89) | 368.59 (206.80, 491.03) | 208.50 (104.12, 407.89) | 290.35 (165.57, 491.03) | 221.94 (124.53, 399.36) | 401.85 (254.45, 572.23) | 221.94 (126.44, 356.73) | 353.00 (150.26, 572.23) | 217.59 (114.01, 419.71) | 355.11 (163.65, 545.34) | |
| Kynu  (nM) | 1.45 (0.92, 2.32) | | 1.77 (0.69, 2.79) | 1.34 (0.85, 2.32) | 1.60 (0.93, 2.70) | 1.53 (0.93, 2.52) | 1.49 (0.88, 3.85) | 1.56 (1.02, 2.37) | 1.39 (0.88, 3.02) | 1.31 (0.79, 2.00) | 2.31 (1.23, 4.21) | 1.24 (0.82, 1.99) | 1.86 (1.13, 4.21) | 1.13 (0.79, 1.77) | 2.37 (1.29, 4.31) | |
| Tau | 349.25 (228.76, 739.39) | | 683.82 (262.42, 1629.67) | 489.96 (243.29, 888.24) | 332.69 (226.15, 921.34) | 309.96 (220.89, 802.55) | 689.70 (295.60, 939.01) | 293.50 (204.45, 966.13) | 417.34 (256.31, 914.49) | 375.44 (239.24, 760.53) | 440.63 (234.96, 939.01) | 284.47 (205.37, 559.90) | 714.38 (290.98, 1572.14) | 288.29 (220.89, 500.60) | 714.38 (282.96, 1329.01) | |
| Biomarker levels presented as untransformed medians (IQR). Biomarker concentrations in pg/mL unless otherwise noted.  Differences in biomarker levels within each complication analyzed using Wilcoxon rank-sum test. Differences significant following adjustment for multiple comparisons using Bonferroni correction (30 comparisons, p<0.001667) shaded. | | | | | | | | | | | | | | | | |
